# Faster poleward range shifts in moths with more variable colour patterns

**DOI:** 10.1038/srep36265

**Published:** 2016-11-03

**Authors:** Anders Forsman, Per-Eric Betzholtz, Markus Franzén

**Affiliations:** 1 Center for Ecology and Evolution in Microbial Model Systems, EEMiS, Department of Biology and Environmental Science, Linnaeus University, SE-391 82 Kalmar, Sweden; 2 Department of Biology and Environmental Science, Linnaeus University, SE-391 82 Kalmar, Sweden; 3 Department of Community Ecology, UFZ, Helmholtz Centre for Environmental Research, Halle, Germany

## Abstract

Range shifts have been documented in many organisms, and climate change has been implicated as a contributing driver of latitudinal and altitudinal range modifications. However, little is known about what species trait(s) allow for faster environmental tracking and improved capacity for distribution expansions. We used data for 416 species of moths, and show that range limits in Sweden have shifted to the north by on average 52.4 km per decade between 1973 and 2014. When also including non-expanding species, average expansion rate was 23.2 km per decade. The rate of boundary shifts increased with increasing levels of inter-individual variation in colour patterns and decreased with increasing latitude. The association with colour patterns indicate that variation in this functionally important trait enables species to cope with novel and changing conditions. Northern range limits also increased with average abundance and decreased with increasing year-to-year abundance fluctuations, implicating production of dispersers as a driver of range dynamics. Studies of terrestrial animals show that rates of poleward shifts differ between taxonomic groups, increase over time, and depend on study duration and latitude. Knowledge of how distribution shifts change with time, location, and species characteristics may improve projections of responses to climate change and aid the protection of biodiversity.

Biodiversity is challenged by environmental modifications brought about by altered land use and climate change^1^,^2^. To enable prognostications of responses to changes in the environment, it is necessary to identify species traits that allow for adaptation to new conditions or enable rapid modifications of range distributions^3^. The rates of range boundary shifts are influenced by dispersal capacity and establishment success. Terrestrial animals, such as birds and insects^4^,^5^,^6^, especially butterflies and moths^4^,^7^,^8^, have proven useful for exploring range dynamics. Butterflies and moths are active flyers, ectothermic, have short generation times and high fecundity, and can swiftly shift their ranges in response to altered conditions^9^. Moths exert top-down effects on their host-plants, include species that constitute pests in agriculture and forestry, and may induce bottom-up effects on their insectivorous predators^10^,^11^. Moths that establish outside their natural distribution range and alter community compositions can therefore disrupt ecosystem functioning^12^,^13^,^14^. However, little is known about why the pace of range shifts varies considerably among studies and among different species^4^,^9^,^15^. A potentially informative trait in this regard is the level of intraspecific variation in colour pattern.

Colour pattern is a functionally important trait. It can influence both predation risk^16^ and the capacity for temperature regulation^17^. Furthermore, colour patterns are genetically and developmentally associated with other phenotypic dimensions, such as body size, disease resistance, reproductive life-history traits, microhabitat use and diet^18^,^19^. Because of the associations of coloration with several other traits, it has been hypothesized that higher levels of inter-individual variation in colour pattern can be considered a proxy of ecological generalization that enables populations and species to utilize broader niches^18^, and that colour polymorphism may therefore allow for more efficient utilization of diverse resources, reduce vulnerability to environmental change, contribute to higher and more stable population densities, which in turn should promote establishment success, and reduce extinction rate^18^,^20^. Variable coloration can also protect prey populations against predation^21^. As a consequence, species with variable colour patterns should be capable of faster range expansions compared with species that do not vary in colour pattern, due to mechanisms operating on colour pattern as such, as well as to processes involving traits that are associated with colour pattern^18^,^20^.

Here, we first evaluate the role of variable colour patterns for range expansions using data for 416 non-migratory species of moths. We compile information for each species on the northward range boundaries in Sweden both in 1973 and in 2014. Next, we investigate whether differences in rates of northward range shifts during this period are associated with variation among species in the level of inter-individual variation in colour pattern, or with host plant niche breadth, duration of flight activity period, dispersal capacity (as estimated by wingspan^22^), overwintering stage^8^, or year-to-year abundance fluctuations^23^. We also examine whether the expected influence of variable colour pattern remains when the potential effect of these other traits is accounted for. Finally, we use information from previous studies of terrestrial animals to compare rates of range shifts between taxonomic groups, and investigate whether and how they vary over time and with latitude.

## Results and Discussion

### Northern range limits

**Figure 1:**
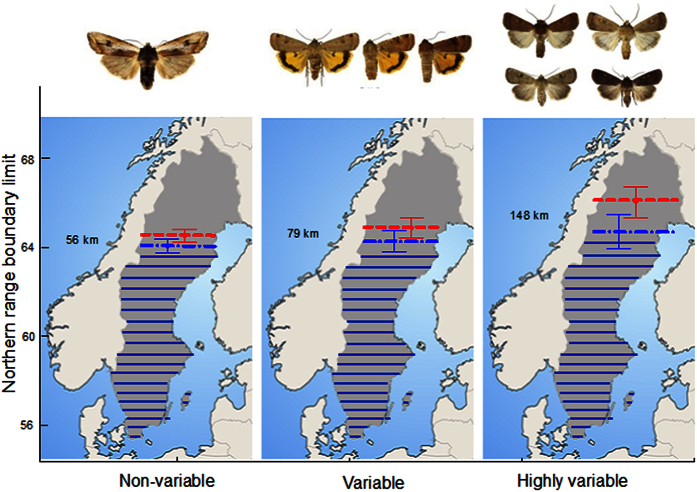
Northern distribution range boundaries in Sweden in 1973 and 2014 for species of moths with non-variable, variable, or highly variable colour patterns. Least-squares means ± SE (obtained from a MIXED model ANOVA) for 1973 (blue, dash dot) and 2014 (red, short dash). Result for 1973 (effect of family: *F*_2,208_ = 0.34, *P* = 0.56; effect of colour pattern: *F*_2,400_ = 0.49, *P* = 0.61). Result for 2014 (family: *F*_1,201_ = 0.88, *P* = 0.348; colour pattern: *F*_2,396_ = 2.41, *P* = 0.091). Numbers indicate mean expansion between 1973 and 2014. The map was generated in Adobe Photoshop CS5 Extended, version 12.0.4 × 32, http://www.adobe.com/se/products/photoshop.html. The image was modified from a base map available under non-restrictive creative commons license obtained from https://commons.wikimedia.org/wiki/File:Scandinavia-template.png. Photos (by Vladimir S. Kononenko) show (from left to right) *Xylena vetusta, Noctua orbona*, and *Xestia xanthographa* representing each category of colour pattern variation.

Our results show that northerly range limits in 1973 were independent of the level of variation in colour pattern (Fig. 1). In 2014, range limits tended to be more towards the north in species with more variable colour patterns (Fig. 1). More than one third (39%, 161 of 416) of the moth species had shifted their northern boundaries by on average 215 ± 166SD km (range 2**–**950 km) from 1973–2014, corresponding to an expansion rate of 52.4 ± 40.6SD km per decade (range 0.5**–**232 km per decade). When non-expanding species were included in the estimate, average expansion rate decreased to 23.2 ± 2.03SD km per decade (paired *t*-test: *t* = 11.51, *n* = 416, *P* < 0.0001).

### Associations of species traits with rates of northerly range expansions

**Figure 2:**
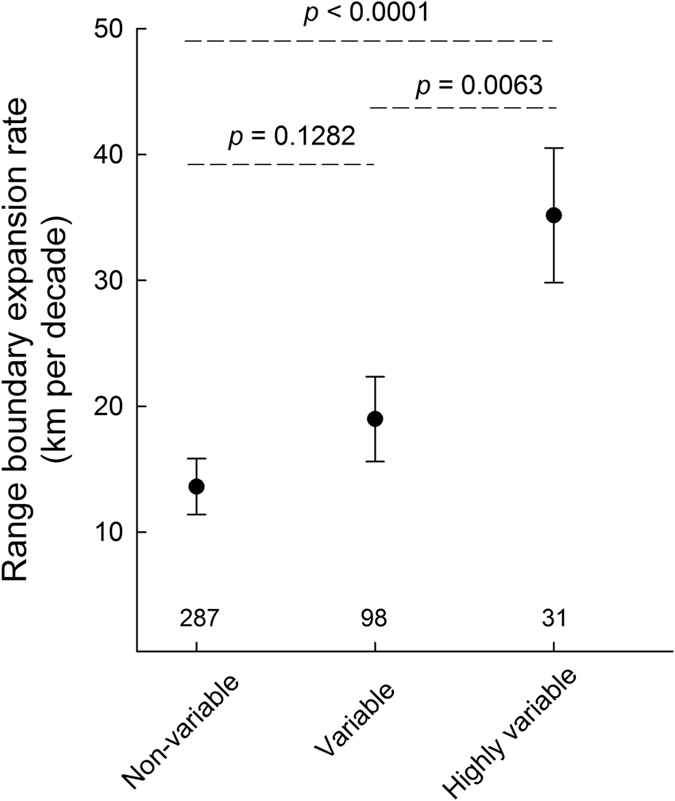
Rates of northward range boundary shifts during the period 1973 to 2014 for species of moths with non-variable, variable, or highly variable colour patterns. Least-squares means ± SE (as obtained from a MIXED model ANOVA). Results, effect of colour pattern: *F*_2,401_ = 8.16, *P* = 0.0003; effect of family: *F*_1,205_ = 2.44, *P* = 0.12, fixed effect of northern boundary in 1973: *F*_1,406_ = 103.61, *P* < 0.0001. The association of faster expansion rates with increasing colour pattern variation did not differ between family Erebidae and Noctuidae (effect of interaction: *F*_2,397_ = 0.67, *P* = 0.51). *P*-values above dashed lines represent statistical significance of differences of least-squares means. Local and overall effect sizes^39^,^40^ (Cohen’s *f*^2^ = 0.027, Cohen’s *d* = 0.33) indicated that the magnitude of the effect of colour pattern variation was small to medium. Values above horizontal axis denote number of species.

Expansion rate decreased with increasing latitude, and increased with increasing variation in colour pattern (Fig. 2). The association of faster expansion rate with increasing variation in colour patterns was evident also when we used a more conservative estimate of range expansion (Supplementary Table S1), and when those species that already occurred in the northernmost part of Sweden in 1973 were omitted (Supplementary Table S2, Supplementary Fig. S1). Results were robust to inclusion of non-resident moth species (Supplementary Table S3).

Expansion rate was associated with overwintering stage (Table 1). Species that overwinter as pupa had expanded northward at a lower rate (Least-squares means as obtained from a MIXED model ANOVA = 15 km per decade, 95% CI = 7–23, *n* = 113) than species that overwinter as eggs (28, 20–36, *n* = 84), but not compared with species that overwinter as larva (23, 18–30, *n* = 211) or as adults (28, 11–46, *n* = 15). The effect size for overwintering stage was small compared with that for variable coloration (Table 1). Rates of range shifts were not associated with any of the other species characteristics that have been proposed to influence the capacity for range expansions, namely host plant niche breadth, length of flight activity period, or dispersal capacity (Table 1). Furthermore, the association of faster range shifts with more variable colour patterns remained also after the potential influences of the other species traits (including overwintering stage) were statistically accounted for (Table 1). These findings implicate inter-individual variation in colour pattern as an important determinant of range shift responsiveness in moths, in accordance with predictions from theory^18^.

The northward range shifts demonstrated here represented new establishments at the northern boundary of the species distributions. The knowledge of the moth fauna in Sweden since 1950 is outstanding. Distribution records by province indicated that northward range shifts generally resulted from sequential establishments of new populations rather than from long-distance movements and founding of isolated northern populations disjunct from the core distribution (Supplementary Fig. S2). These may have stemmed from effects that were mediated by colour pattern, or from indirect effects of traits associated with colour pattern^18^,^19^, on either dispersal capacity or net colonisations.

Neither the duration of the flight period nor wing span, which is a satisfactory proxy for dispersal ability of lepidopterans in large scale analyses^22^, was associated with expansion rate (Table 1). There is experimental and comparative evidence from a broad range of taxonomic groups that species with more variable colour patterns have larger range sizes, higher establishment success, and lower extinction risk^20^,^24^,^25^,^26^,^27^. However, we are not aware of any previous demonstration that species with more variable colour patterns have undergone faster range shifts.

The shifts in northerly range limits could partly have reflected a latitudinal bias in sampling effort, for example if sampling was more intense in the northern parts of Sweden during 1974–2014 than before 1973. Such sampling bias, if present, might have resulted in that rates of expansion were overestimated, but it would not generate a spurious association of expansion rate with variable colour pattern.

### Association of northern boundary limits with population dynamics

To evaluate whether species distributions were influenced by population dynamics, range limits were analysed in relation to abundance data obtained from a long-term (2003–2013) monitoring study^23^. Northerly range limits decreased with increasing year-to-year changes in abundance (1973: *r* = −0.25, *n* = 236, *P* = 0.0001; 2014: *r* = −0.26, *n* = 240, *P* < 0.0001), suggesting that a high ability to cope with environmental change allows for more northerly distributions, and that poorly buffered species cannot readily establish in areas with harsh climate conditions. Northern boundaries also increased with increasing average abundance (1973: *r* = 0.22, *n* = 236, *P* = 0.0008; 2014: *r* = 0.22, *n* = 240, *P* = 0.0005), perhaps reflecting that species with high population densities produce more long-distance dispersers, or are less likely to go extinct once established^20^,^27^.

### Associations of rates of poleward shifts with latitude, taxonomic group, study period and study duration

**Figure 3:**
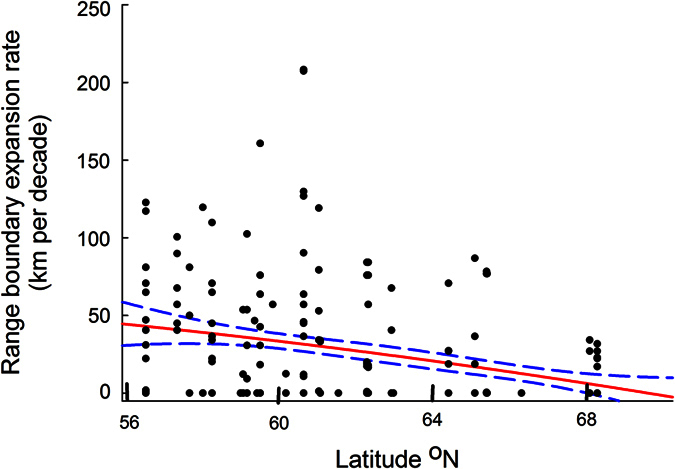
Rates of northward range boundary shifts of moths in Sweden decrease with increasing latitude. Figure show rates of northern range boundary expansion rate (in km per decade) during the period 1973 to 2014 in relation to the northern boundary limit in 1973. Red line represents least squares linear regression (*F*_1,334_ = 47.18, *R*^2^ = 0.12, *P* < 0.0001, *r*_s_ = −0.43, *n* = 336, *P* < 0.0001). Dashed blue lines indicate 95%CI.

Northerly expansion rates of moths decreased with increasing latitudes (Table 1, Fig. 3). This might indicate that poleward range shifts were limited more by lack of suitable habitats, resources and temperature conditions than by poor dispersal capacity. It is possible that an effect of latitude, as indicated by our results, may have contributed to the heterogeneity of estimated rates of range expansions seen among studies. Assuming that northerly range shifts are driven partly by climate change^6^,^28^, and that the speed of temperature change varies among biomes^29^ and increases over time^30^, one would hypothesize rates of range shifts to differ among studies carried out during different periods and in different regions - if species are keeping pace with moving climates.

**Figure 4:**
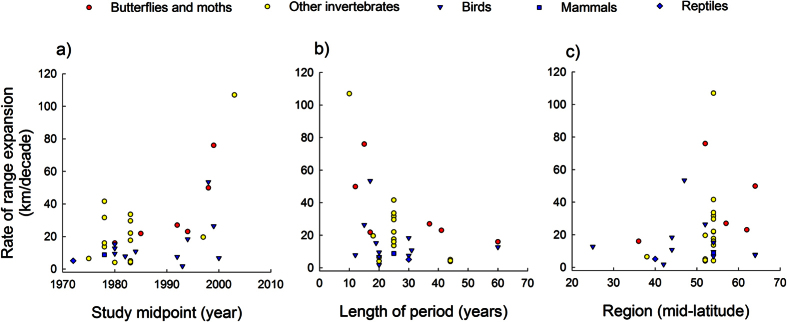
Rates of range boundary expansions vary among groups of terrestrial animals and change over time and with latitude. Rates of boundary shifts for butterflies and moths (*N* = 7 studies), invertebrates other than butterflies and moths (*N* = 14), reptiles (*N* = 1), birds (*N* = 11), and mammals (*N* = 1) were obtained from previous studies (see Supplementary Table S4). Results from statistical evaluation of associations are reported in Table 2.

Comparisons of rates of latitudinal range margin shifts for different groups of terrestrial animals reported in previous studies (Supplementary Table S4) partly support the above predictions. Overall, invertebrates have shifted their leading-edge margins poleward at faster rates compared with vertebrates, and the rates of range shifts have been faster in studies conducted over shorter intervals and during more recent time periods (Table 2, Fig. 4a,b). Rates of range margin shifts also tended to decrease with increasing latitude (Table 2, Fig. 4c).

The changes over time and with latitude were stronger (as evidenced by comparisons of effect sizes) when the analysis was restricted to data for butterflies and moths (Table 2). The variation in rates of range shifts among species of moths within our study (0–232 km per decade) exceeded the variation in mean values among studies (3.9–76 km per decade, Supplementary Table S4). This finding corroborates the conclusion from previous reviews that there is greater variation among species within taxonomic groups than between groups^6^. In this context, it is encouraging that mid-latitude, sampling period midpoint, and sampling duration accounted for 99% of the total variation in expansion rate among studies (Table 2). Despite the strength of the associations these last findings must be interpreted with caution. Because of the small sample sizes and inter-correlations between external explanatory variables, firm conclusions regarding the nature of the association with latitude across studies require further investigations.

## Conclusions

In conclusion, our study shows that estimates of rates of range expansion differ between taxonomic groups and are influenced by a combination of study characteristics (time period and duration), environmental factors (latitude), and species attributes (colour pattern variation). This should benefit attempts aimed at projecting responses to future climate change in the form of geographic distribution shifts, alterations in community composition and how this might influence ecosystem functioning. The association with latitude, together with the lack of effect of host plant niche width, suggest that expansions from southern, biologically diverse, regions to northern depauperate areas have been influenced more by environmental factors than by species interactions. That more variable colour patterns have been associated with faster northerly range expansions provides rare evidence, in agreement with predictions from theory, that populations and species harbouring higher levels of individual variation in functionally important traits are better able to cope with novel and changing environmental conditions. That species of moths with higher average abundances and less pronounced year-to-year fluctuations have shifted their leading-edge margins further northward implicates production of dispersers and tolerance to environmental change as drivers of range dynamics. The realization that information on species traits that can be readily obtained at low cost might serve as a proxy for responsiveness and help identifying which species are more or less prone to become endangered or invasive is promising for the protection of biodiversity.

## Methods

### Distribution limits and estimation of northward range boundary shifts

Information on range distributions and expansion distances was obtained from the national province level catalogue of Swedish butterflies and moths (Catalogus Lepidopterorum Sueciae, http://www2.nrm.se/en/catalogus.html.se, accessed November 15, 2015). In our main approach, range expansion was measured as the distance (km) between the northern range boundary limit of the province occupied in 1973 and the northern range limit of the northernmost province occupied in 2014 (see Fig. 1 in Betzholtz *et al*.^7^). For species that colonized Sweden during the study period, we used the southern limit of the colonized province as the northern range margin in the analyses. Geographical limits of the 30 Swedish provinces were extracted from maps. Provinces in Sweden vary in shape and size, and using the northern margin of the northernmost province might result in overestimation of range expansions for species that had established north of the southern margin in a new province but that had not spread throughout the entire province. To evaluate whether our results and conclusions were influenced by this potential bias we also calculated a conservative estimate of range expansion, as the distance (km) between the northern range limit of the northernmost province occupied in 1973 and the southern margin of the northernmost province occupied in 2014 (see Fig. 1 in Betzholtz *et al*.^7^). Using the conservative estimate in the analyses resulted in that the exact parameter values of the statistical tests changed somewhat but these minor changes did not in any way influence the results or conclusions regarding the rate of northward range expansions in relation to colour pattern variability (Supplementary Table S1).

### Study species, classification of colour pattern diversity, host plant specificity, flight activity period, wingspan, and overwintering stage

Two authors (PEB and MF) performed expert classifications^23^, independently of each other and blind with regard to range distribution data, of inter-individual colour pattern variability of all moth species included in the study. Species within which there is no apparent variation among individuals in coloration and wing pattern were classified as non-variable; species in which individuals vary either in the size, shape, or coloration of colour pattern elements were classified as variable; and species in which individuals vary considerably in size, shape, and coloration of pattern elements or with regard to presence/absence of pattern elements were classified as highly variable^23^ (Fig. 1). Species that were sexually dichromatic were classified as having variable coloration only if variation was manifest within one or both sexes, otherwise they were considered non-variable. The independent classifications diverged for 20 (4.8%) of the 416 non-migratory species and for 23 (4.7%, 7 species with non-variable colouration, 15 with variable coloration, and 1 with highly variable coloration) of all 490 species, and in these cases we consulted Skou^31^ and checked species description texts and corresponding figures before the final classification was made. The classification of species with regard to colour pattern variability used in the analyses (Supplementary Table S5) was thus very robust.

Moths differ in the degree of host-plant specificity and species with narrower feeding niches may have lower capacity for range expansions^7^,^32^,^33^. If species that have monophagous larvae also have less variable colour patterns as adults, an association of range expansion with variable coloration might be driven by a confounding underlying effect of host-plant specificity, rather than by mechanisms that involve coloration. To evaluate and statistically control for this possibility, all species were classified for niche breadth as belonging to one of three categories of larval host-plant specificity: monophagous species that feed only on a single plant species, oligophagous species that feed on a few plants species (use between two and five species or restricted to a particular plant genera/family), and polyphagous species that feed on six or more different plant species or genera^7^.

It can be hypothesized that species with longer adult flight activity periods have a higher capacity for long distance dispersal and range expansions^22^. To evaluate this possibility, we extracted information on duration of adult flight activity period for all species according to Svensson^34^. The results of a meta-analysis suggest that wingspan can be used a satisfactory proxy for butterfly dispersal ability in large scale analyses^22^. To further evaluate whether the rate of range expansions may have been influenced by dispersal capacity, we extracted information on wingspan (mm), according to Skou^31^ and Emmet^35^. We used data for male size, but because male and female size is strongly correlated this is unlikely to affect our results and conclusions. Three (<1%) of the 490 moth species in our data set had flightless females (three *Orgyia* spp.), but it is highly unlikely that this compromised the dispersal distance comparison.

Data on butterflies from Finland suggests that overwintering stage is correlated to range expansion, with adult over-winterers showing the largest increase in northern range limit, although with the significance of overwintering stage disappearing when the effects of other ecological predictor variables were accounted for^8^. To evaluate whether the rate range expansions of moths also may have been influenced by overwintering stage, we extracted information on overwintering life-history stage (egg, larva, pupa or imago) from Skou^31^ and Svensson^34^.

To evaluate whether species distributions were influenced by population dynamics, range limits were analysed in relation to average abundance and the magnitude of year-to-year abundance fluctuations. Abundance data, available for 246 moth species, was obtained from a long-term (2003–2013) monitoring study carried out near the village of Böste (55°20′37″N, 13°18′54″E) in the southernmost part of Sweden^23^. Average abundance was calculated for each species across the 11 sampling years, and abundance fluctuations were quantified using CV of abundance over years^23^. Average abundance was log-transformed prior to analysis. Abundance data was available for only a subset of all moth species included in this study.

For the analyses and results reported here we used the systematics of Karsholt and Stadel Nielsen^36^. A list of species, along with information on colour pattern variability and northern range limits, included in our data set is available in the Supplementary Table S5.

### Comparisons of rates of latitudinal range margin expansions among taxonomic groups, over time and with latitude

We examined whether and how estimates of rates of poleward range expansions vary among different studies depending on taxonomic group, and change with time and latitude. To this end, the ISI Web of Knowledge (Science Citation Index expanded 1945-present) database was searched December 4, 2015, using the search string: geographic AND (distribution* OR range* OR boundary) AND (shift* OR limit* OR expansion) AND species AND (northward OR northern OR poleward OR southward OR southern) [an asterisk denotes a wild card search term allowing for several permutations of each intervention type]. The citation map function in Web of Knowledge, forward and backward, was used to find additional studies. To control for positive publishing bias, we extracted data only from multi-species studies that reported information for four or more different species of terrestrial animals. Average estimates across species were obtained for butterflies and moths (*N* = 7 studies), invertebrates other than butterflies and moths (*N* = 14), reptiles (*N* = 1), birds (*N* = 11), and mammals (*N* = 1) (see Supplementary Table S4).

### Statistical analysis

To test if northern range limits in 1973 and 2014 were associated with colour pattern diversity while statistically adjusting for variation among genera we performed general linear mixed models implemented using the procedure MIXED in SAS^37^,^38^. We analysed data for 1973 and 2014 separately. We treated the northern range boundary limits as the dependent variable, colour pattern (non-variable, variable or highly variable) as a fixed factor with three levels, family as a fixed factor with two levels, and genus was included as a random factor to account for greater similarity among more closely related species. Including family and genus as explanatory variables in the statistical analyses partially accounts for greater similarity than expected by chance in response variables and ecological traits among species that are on average more closely related, although without completely removing effects of similarity owing to shared ancestry. The mixed model approach was used also to test if the rate of northward range expansion was associated with the degree of inter-individual variation in colour pattern. Log-transformed range expansion was treated as the dependent variable, colour pattern as a fixed factor with three levels, family as a fixed factor with two levels, and genus was included as a random factor. In this last analysis, log-transformed northern range boundary limit in 1973 was included as a covariate to take into account that the potential for northward range expansions decreased with increasing northern range boundaries. Data on log-transformed range expansion did not violate the assumption of homogeneity of variances (Levene’s test, *F*_2, 420_ = 2.29, *P* = 0.1027).

To evaluate effects of other species traits that potentially may influence range boundaries and the rate of range expansions, and to examine whether the associations with variable coloration remained statistically significant when the contribution of other potential sources of variation were statistically accounted for, we fitted additional models that also included duration of adult activity period as a fixed covariate, wingspan as a fixed covariate, larval niche breadth as a fixed effect with three levels, and overwintering stage as a fixed effect with four levels. The Kenward-Roger method was used to approximate degrees of freedom^38^. Statistical significance of fixed effects was assessed using *F* statistic in the Type 3 test for fixed effects^37^. Statistical significance of random factors can be assessed using the Wald *Z* test and using the Log-likelihood ratio test with one degree of freedom per random effect^37^,^38^. However, we included genus (and family) in the models to control for any variation in the dependent variables that might be due to relatedness. We were not interested in evaluating or quantifying effects of genus as such, and formal testing for random effects of genus was not necessary for evaluating the main hypotheses under investigation. Parameter estimates and statistical significance levels associated with genus are therefore not reported. To quantify the magnitude of main effects, we estimated local effect sizes (Cohen’s *f*^2^ and *η*^2^)^39^,^40^, as well as overall effects size (by converting the Pearson correlation coefficient between rate of range expansion and colour pattern variation to Cohen’s *d*)^39^.

## Additional Information

**How to cite this article**: Forsman, A. *et al*. Faster poleward range shifts in moths with more variable colour patterns. *Sci. Rep.* **6**, 36265; doi: 10.1038/srep36265 (2016).

**Publisher’s note:** Springer Nature remains neutral with regard to jurisdictional claims in published maps and institutional affiliations.

## Supplementary Material

Supplementary Information

## Figures and Tables

**Table 1:** Associations of rates of range shifts in moths.

Source of variation	*df*	estimate ± SE	*Cohen’s *f*^2^	**Eta-squared *η*^2^	*F*	*P*
Family	1, 163	−0.22 ± 0.281	0.0053	—	0.62	0.4316
Colour pattern variation	2, 391	−1.24 ± 0.386	0.0288	0.0102	5.27	0.0055
		−0.96 ± 0.416				
Northern range limit 1973	1, 390	−21.92 ± 1.70	0.4312	0.1409	166.50	<0.0001
Host plants used	1, 323	0.285 ± 0.168	0.0037	0.0010	2.86	0.0916
Wing span	1, 203	−0.024 ± 0.013	0.0120	0.0010	3.38	0.0674
Adult activity period	1, 402	−0.029 ± 0.043	0.0025	0.0001	0.45	0.5043
Overwintering stage	3, 140	−0.071 ± 0.664	0.0068	0.0031	3.88	0.0106
		−0.611 ± 0.639				
		−1.140 ± 0.642				

Results from general linear mixed model analysis of variance (GLMM) for effects of inter-individual variation in colour pattern, northward range boundary in 1973, niche breadth (host plants used), dispersal capacity (wing span), duration of adult flight activity period (weeks), and overwintering stage (egg, larva, pupa or imago) on rates of northward range boundary shifts in species of non-migratory moths in Sweden during the past 41 years (1973–2014).

*df* represents numerator and denominator degrees of freedom.

^*^According to Cohens^39^ guidelines, *f *^2^ ≥ 0.02, *f*^ 2^ ≥ 0.15, and *f*^ 2^ ≥ 0.35 represent small, medium, and large effect sizes, respectively.

^**^Eta-squared, *η*^2^, was estimated from a GLM model in which genus was treated as a fixed rather than as a random effect.

**Table 2:** Associations across studies of range expansion rate with organism type, study period, and latitude.

Source of variation	*df*	estimate ± SE	*Eta-squared *η*^2^	*F*	*P*
*All studies*: Model *F*_4,29_ = 7.43, *P* = 0.0003, *R*^*2*^ = 0.51					
Organism type	1	19.07 ± 6.26	0.159	9.29	0.0049
Study midpoint (year)	1	1.35 ± 0.367	0.232	13.61	0.0009
Study duration (years)	1	−0.67 ± 0.281	0.096	5.67	0.0241
Mid-latitude	1	−0.64 ± 0.432	0.037	2.18	0.1503
*Butterflies and moths*: Model *F*_3,3_ = 1402.13, *P* < 0.0001, *R*^*2*^ = 0.99					
Study midpoint (year)	1	3.46 ± 0.079	0.456	1918.50	0.0001
Study duration (years)	1	−0.59 ± 0.029	0.098	412.39	0.0003
Mid-latitude	1	−2.04 ± 0. 60	0.279	1172.98	0.0001

Results from general linear model analyses of variance for effects of organism type (vertebrate *versus* invertebrate), midpoint (calendar year) of study period used to estimate range expansion, duration of study period (number of years), and latitudinal midpoint of study region on average rates of latitudinal range boundary shifts as reported in other studies (Supplementary Table S4). Top of table shows results based on estimates from all studies.

Bottom of table shows results based on analysis of data for butterflies only.

*df* represents nominator degrees of freedom.

All interactions were non-significant (all *P* > 0.10) and removed from the models.

^*^Eta-squared, *η*^2^, is a measure of local effect size^39^.
