# Supplementary material for: Faster poleward range shifts in moths with more variable colour patterns
Source: Sci Rep. 2016 Nov 3;6:36265. doi: 10.1038/srep36265 (PMC5093557; doi:10.1038/srep36265)
Supplement: Supplementary Information [file srep36265-s1.pdf]

Faster poleward range shifts in moths with more variable colour patterns

Anders Forsman[a*], *Per-Eric Betzholtz*[b], *Markus Franzén*[c]

Supplementary Information

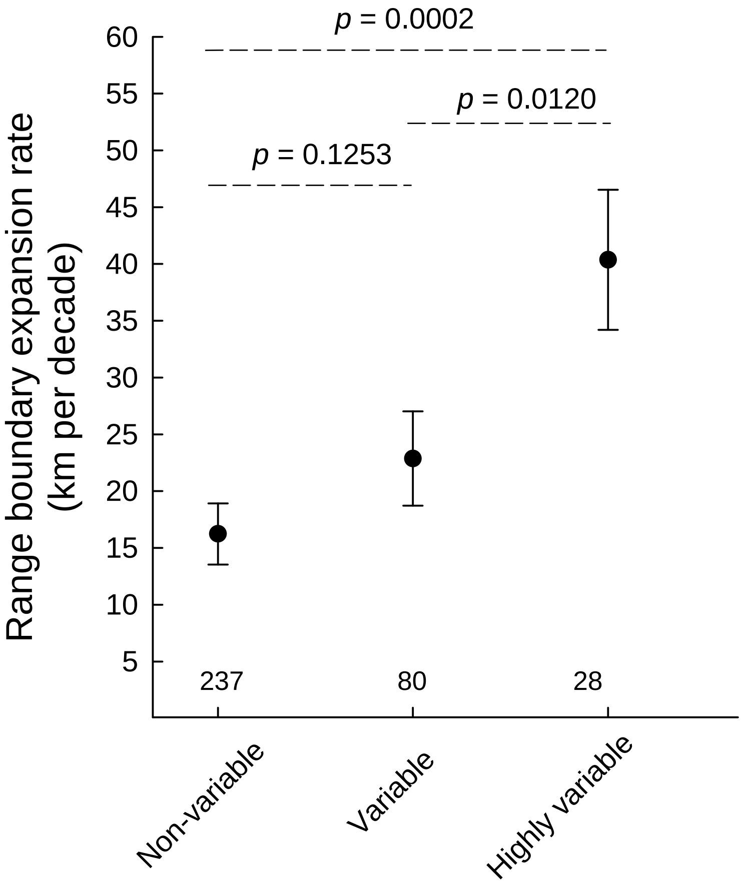

Supplementary Figure S1. Rates of range boundary shifts based on the data set in which the northernmost species were not included. Rates of northward range boundary shifts during the period 1973 to 2014 for species of moths with non-variable, variable, or highly variable colour patterns. Figure shows Least-squares means ± SE (as obtained from a MIXED model ANOVA). Results are based on a data set that did not include those species that occurred in the northernmost part of Sweden in 1973. *P*-values above dashed lines represent statistical significance of differences of least-squares means. Values above horizontal axis denote number of species.

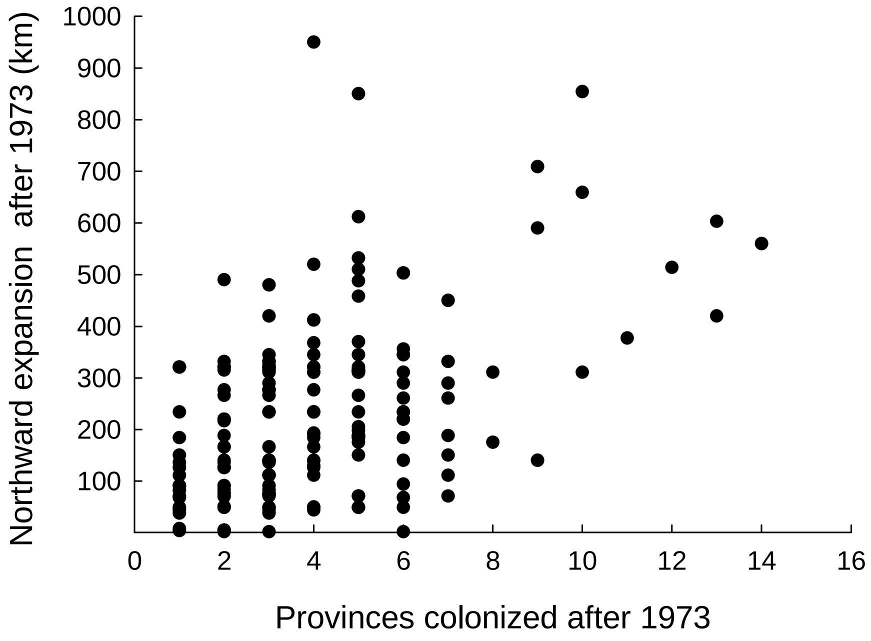

Supplementary Figure S2. Association of magnitude of northward range expansions with number of newly colonized provinces. Data for species of moths belonging to family Noctuidae and Erebidae that expanded northwards in Sweden during the period 1973 to 2014. The positive association (*r* = 0.48, *n* = 188, *P* < 0.0001), together with the lack of observations in the upper left corner of the graph, indicate that northward range shifts generally resulted from sequential establishments of new populations rather than from long-distance movements and founding of isolated northern populations disjunct from the core distribution.

Supplementary Table S1. **Results based on the more conservative estimate of range expansion**

Source of variation	*df*	estimate ± SE	*F*	*P*
Family	1, 209	-0.26 ± 0.210	1.54	0.2158
Colour pattern variation	2, 399	-0.85 ± 0.291	5.22	0.0058
		-0.49 ± 0.317		
Northern range limit 1973	1, 402	-13.32 ± 1.218	119.51	< 0.0001

Results from general linear mixed model analysis of variance for effects of family, inter-individual variation in colour pattern and northward range boundary in 1973 on rates of northward range boundary shifts in species of non-migratory moths in Sweden during the past 41 years (1973 – 2014).

Analysis was performed on data on the conservative estimate of expansion rate based on the southern (rather than the northern) margin of the northernmost province occupied in 2014.

df represents numerator and denominator degrees of freedom.

Supplementary Table S2. Results of a reduced data set in which the northernmost species were not included

Source of variation	*df*	estimate ± SE	*F*	*P*
Family	1, 179	-0.31 ± 0.35	0.80	0.3724
Colour pattern variation	2, 331	-1.35 ± 0.444	4.88	0.0081
		-0.96 ± 0.485		
Northern range limit 1973	1, 338	-20.0 ± 2.06	94.10	< 0.0001

Results from general linear mixed model analysis of variance for effects of family, inter-individual variation in colour pattern and northward range boundary in 1973 on rates of northward range boundary shifts in species of non-migratory moths in Sweden during the past 41 years (1973 – 2014).

Analysis was performed on a reduced data set in which species that occurred in the northernmost part of Sweden in 1973 were not included.

df represents numerator and denominator degrees of freedom.

Supplementary Table S3. **Results based on analysis of an expanded data set that included also non-resident, migratory species**

Source of variation	*df*	estimate ± SE	*F*	*P*
Family	1, 201	-0.32 ± 0.282	1.31	0.2534
Migration status	1, 447	-1.33 ± 0.377	12.36	0.0005
Colour pattern variation	2, 438	-1.33 ± 0.382	6.69	0.0014
		-0.88 ± 0.41		
Northern range limit 1973	1, 445	-21.09 ± 1.58	174.24	< 0.0001

Results from general linear mixed model analysis of variance for effects of family, migration status, inter-individual variation in colour pattern, and northward range boundary in 1973 on rates of northward range boundary shifts in species of migratory and non-migratory moths in Sweden during the past 41 years (1973 – 2014).

df represents numerator and denominator degrees of freedom.

Supplementary Table S4. **Comparison of estimated mean rates of latitudinal range margin shifts for species in different groups of terrestrial animals reported in previous studies**

Organisms	No. species	Sampling mid-point	Number of years	Rate of range shift km/decade	Region	Mid-latitude	Study
Invertebrates							
Butterflies	29	1985	17	21.8	Great Britain	54°N	[1]
Butterflies	48	1998	12	49.9	Finland	64°N	[2]
Butterflies	181	1999	15	76	Europe	52°N	[3]
Moths	282	1992	37	27	Sweden	57°N	[4]
Butterflies	41	1980	60	16	South Korea	36°N	[5]
Butterflies	61	1983	44	3.9	Netherlands	52°N	[6]
Moths	416	1994	41	23.2	Sweden	62°N	*This study*
Grasshoppers	22	1978	25	13.6	Great Britain	54°N	[1]
Dragonflies	20	1978	25	41.6	Great Britain	54°N	[1]
Lacewings	6	1983	25	17.6	Great Britain	54°N	[1]
Woodlice	8	1978	25	31.6	Great Britain	54°N	[1]
Ground beetles	59	1983	25	22	Great Britain	54°N	[1]
Harvestmen	4	1980	20	4	Great Britain	54°N	[1]
Longhorn beetles	11	1978	25	16	Great Britain	54°N	[1]
Dragonflies	90	1997	18	19.6	Europe	52°N	[7]
Insects	4	1975	20	6.4	Japan	38°N	[8]
Bees	207	1983	44	5	Netherlands	52°N	[6]
Hoverflies	202	1983	44	4.3	Netherlands	52°N	[6]
Gall wasps	4	2003	10	107	Great Britain	54°N	[9]
Spiders	85	1983	25	33.6	Great Britain	54°N	[1]
Millipedes	6	1983	25	29.6	Great Britain	54°N	[1]
Vertebrates							
Birds	59	1980	20	9.5	Great Britain	54°N	[10]
Birds	40	1994	30	18.4	North America	44°N	[11]
Birds	150	1982	12	7.8	Finland	64°N	[12]
Birds	22	1980	19	15.3	Great Britain	54°N	[1]
Birds	55	1984	31	10.8	North America	44°N	[13]
Birds	105	1998	17	53.5	France	47°N	[14]
Birds	129	1993	20	1.8	North America	42°N	[15]
Birds	165	1999	15	26.4	Europe	52°N	[3]
Birds	34	1992	30	7.5	Finland	64°N	[16]
Birds	464	1980	60	12.7	Australia	25°S	[17]
Birds	77	2000	20	6.75	Great Britain	54°N	[18]
Mammals	9	1978	25	8.8	Great Britain	54°N	[1]
Reptiles (lizards and snakes)	30	1972	30	5.1	Spain	40°N	[19]

Supplementary Table S5. List of moth species included in the study, and their classification with regard to northern range limits in 1973 and 2014 (Swedish Grid RT 90 coordinate system) and colour pattern variability (0=non-variable, 1= variable, 3= highly variable)

Family	Species	Range limit 1973	Range limit 2014	Colour pattern variability
Erebidae	Acerbia alpina	7670605	7670605	1
Erebidae	Arctia caja	7580662	7580662	2
Erebidae	Arctia villica	6135149	6135149	1
Erebidae	Arctornis l-nigrum	6550795	6550795	0
Erebidae	Atolmis rubricollis	6911078	6915078	0
Erebidae	Callimorpha dominula	6726787	6726787	1
Erebidae	Callistege mi	7670605	7670605	0
Erebidae	Calliteara abietis	6911078	6982078	1
Erebidae	Calliteara pudibunda	6775458	6911458	2
Erebidae	Calyptra thalictri	.	6675586	0
Erebidae	Catocala adultera	7670605	7670605	0
Erebidae	Catocala electa	6268311	6268311	0
Erebidae	Catocala elocata	6268311	6268311	0
Erebidae	Catocala fraxini	7580662	7580662	1
Erebidae	Catocala fulminea	6434180	6937180	1
Erebidae	Catocala nupta	6911078	6982078	1
Erebidae	Catocala pacta	6775458	6775458	0
Erebidae	Catocala promissa	6770907	6775907	0
Erebidae	Catocala sponsa	6726787	6770787	0
Erebidae	Colobochyla salicalis	6359033	6771033	0
Erebidae	Coscinia cribraria	7580662	7580662	1
Erebidae	Cybosia mesomella	7259242	7580242	0
Erebidae	Diacrisia sannio	7580662	7580662	0
Erebidae	Diaphora mendica	6982210	6982210	0
Erebidae	Dicallomera fascelina	7670605	7670605	1
Erebidae	Dysauxes ancilla	6460852	6460852	0
Erebidae	Eilema complana	7580662	7580662	0
Erebidae	Eilema depressa	6987888	6987888	0
Erebidae	Eilema griseola	6600439	6726439	0
Erebidae	Eilema lurideola	7580662	7580662	0
Erebidae	Eilema lutarella	7148468	7148468	0
Erebidae	Eilema pygmaeola	6359033	6636033	0
Erebidae	Eilema sororcula	6460852	6805852	0

Erebidae	Eublemma minutata	6460852	6543852	0
Erebidae	Euclidia glyphica	7670605	7670605	0
Erebidae	Euproctis chrysorrhoea	6562476	6562476	0
Erebidae	Euproctis similis	6775458	6775458	0
Erebidae	Grammia quenseli	7670605	7670605	1
Erebidae	Herminia grisealis	6914655	6914655	0
Erebidae	Herminia tarsicrinalis	6434180	6434180	0
Erebidae	Herminia tarsipennalis	7148468	7148468	0
Erebidae	Holoarctia puengeleri	7670605	7670605	1
Erebidae	Hypena crassalis	7580662	7580662	2
Erebidae	Hypena lividalis	.	6202704	0
Erebidae	Hypena obesalis	.	6434180	2
Erebidae	Hypena proboscidalis	7580662	7580662	1
Erebidae	Hypena rostralis	6726787	7246787	2
Erebidae	Hypenodes humidalis	7670605	7670605	0
Erebidae	Hyphoraia aulica	6726787	6726787	0
Erebidae	Laelia coenosa	.	6268311	0
Erebidae	Laspeyria flexula	6914655	7148655	0
Erebidae	Leucoma salicis	7670605	7670605	0
Erebidae	Lithosia quadra	6726787	6914787	0
Erebidae	Lygephila craccae	6775458	6911458	0
Erebidae	Lygephila pastinum	7148468	7225468	0
Erebidae	Lygephila viciae	6726787	6775787	1
Erebidae	Lymantria dispar	6550795	6600795	0
Erebidae	Lymantria monacha	6911078	6915078	2
Erebidae	Macrochilo cribrumalis	6914655	6914655	0
Erebidae	Miltochrista miniata	6600439	6911439	0
Erebidae	Minucia lunaris	6268311	6359311	0
Erebidae	Nudaria mundana	7580662	7580662	0
Erebidae	Orgyia antiqua	7670605	7670605	0
Erebidae	Orgyia antiquiodes	7580662	7580662	0
Erebidae	Orgyia recens	6911078	6911078	0
Erebidae	Paracolax tristalis	6726787	6914787	0
Erebidae	Pararctia lapponica	7670605	7670605	0
Erebidae	Parascotia fuliginaria	6982210	7259210	0
Erebidae	Parasemia plantaginis	7670605	7670605	1
Erebidae	Pelosia muscerda	6562476	6782476	0
Erebidae	Pelosia obtusa	6460852	6460852	0
Erebidae	Pericallia matronula	.	6309584	1
Erebidae	Phragmatobia fuliginosa	7670605	7670605	0
Erebidae	Phytometra viridaria	7580662	7580662	0
Erebidae	Polypogon lunalis	6770907	6775907	1
Erebidae	Polypogon strigilata	7259242	7259242	1
Erebidae	Polypogon tentacularia	7670605	7670605	0
Erebidae	Prodotis stolida	.	6726787	0
Erebidae	Rhyparia purpurata	6726787	6726787	0
Erebidae	Rivula sericealis	7259242	7259242	1
Erebidae	Schrankia costaestrigalis	6770907	6910907	1

Erebidae	Schrankia taenialis	6460852	6551852	0
Erebidae	Scoliopteryx libatrix	7670605	7670605	0
Erebidae	Setema cereola	7580662	7691662	0
Erebidae	Setina irrorella	7670605	7670605	0
Erebidae	Setina roscida	6460852	6460852	0
Erebidae	Spilosoma lubricipedum	7259242	7261242	2
Erebidae	Spilosoma luteum	6770907	6910907	0
Erebidae	Spilosoma urticae	6726787	6726787	0
Erebidae	Spiris striata	6434180	6434180	1
Erebidae	Thumatha senex	7580662	7580662	0
Erebidae	Trisateles emortualis	6914655	6987655	0
Erebidae	Tyria jacobaeae	6726787	6726787	0
Noctuidae	Abrostola asclepiadis	6726787	6960787	0
Noctuidae	Abrostola tripartita	7259242	7580242	0
Noctuidae	Abrostola triplasia	7148468	7148468	0
Noctuidae	Acosmetia caliginosa	6268311	6268311	0
Noctuidae	Acronicta aceris	7148468	7148468	0
Noctuidae	Acronicta alni	6914655	7259655	2
Noctuidae	Acronicta auricoma	7670605	7670605	1
Noctuidae	Acronicta cuspis	6914655	6914655	0
Noctuidae	Acronicta euphorbiae	7580662	7580662	0
Noctuidae	Acronicta leporina	7580662	7580662	1
Noctuidae	Acronicta megacephala	7670605	7670605	0
Noctuidae	Acronicta menyanthidis	7670605	7670605	0
Noctuidae	Acronicta psi	7259242	7580242	0
Noctuidae	Acronicta rumicis	7580662	7580662	1
Noctuidae	Acronicta strigosa	6268311	6534311	1
Noctuidae	Acronicta tridens	6914655	6914655	0
Noctuidae	Actebia fennica	7580662	7580662	1
Noctuidae	Actebia praecox	7580662	7580662	0
Noctuidae	Actinotia polyodon	7225141	7225141	0
Noctuidae	Agrochola circellaris	7259242	7574242	0
Noctuidae	Agrochola helvola	7260962	7575962	0
Noctuidae	Agrochola litura	6914655	6982655	0
Noctuidae	Agrochola lota	7259242	7580242	0
Noctuidae	Agrochola lychnidis	6395244	6727244	1
Noctuidae	Agrochola macilenta	6770907	7258907	1
Noctuidae	Agrochola nitida	6726787	6910787	0
Noctuidae	*Agrotis bigramma*	.	6434180	1
Noctuidae	Agrotis cinerea	6600439	6600439	1
Noctuidae	Agrotis clavis	7580662	7671662	1
Noctuidae	Agrotis exclamationis	7580662	7580662	1
Noctuidae	Agrotis ipsilon	7225141	7225141	1
Noctuidae	Agrotis puta	.	6202704	1
Noctuidae	Agrotis ripae	6550795	6550795	1
Noctuidae	Agrotis segetum	7225141	7225141	2
Noctuidae	Agrotis vestigialis	7580662	7580662	1
Noctuidae	Allophyes oxyacanthae	6911078	6988078	0

Noctuidae	Ammoconia caecimacula	6914655	6914655	0
Noctuidae	Amphipoea crinanensis	7259242	7261242	1
Noctuidae	Amphipoea fucosa	7580662	7580662	1
Noctuidae	Amphipoea lucens	7580662	7580662	1
Noctuidae	Amphipoea oculea	7580662	7580662	1
Noctuidae	Amphipyra berbera	6726787	6775787	0
Noctuidae	Amphipyra livida	.	6309584	0
Noctuidae	Amphipyra perflua	6600439	7259439	0
Noctuidae	Amphipyra pyramidea	6911078	6982078	0
Noctuidae	Amphipyra tragopoginis	7670605	7670605	0
Noctuidae	Anaplectoides prasina	7259242	7580242	0
Noctuidae	Anarta myrtilli	7580662	7580662	0
Noctuidae	Anorthoa munda	6395244	6600244	0
Noctuidae	Antitype chi	7580662	7580662	0
Noctuidae	Apamea anceps	7148468	7259468	0
Noctuidae	Apamea crenata	7670605	7670605	2
Noctuidae	Apamea epomidion	6359033	6727033	0
Noctuidae	Apamea furva	7580662	7580662	0
Noctuidae	Apamea illyria	7259242	7259242	0
Noctuidae	Apamea lateritia	7670605	7670605	0
Noctuidae	Apamea lithoxylaea	6726787	6775787	0
Noctuidae	Apamea maillardi	7670605	7670605	1
Noctuidae	Apamea monoglypha	7580662	7580662	1
Noctuidae	Apamea oblonga	6911078	6911078	1
Noctuidae	Apamea ophiogramma	6914655	7148655	2
Noctuidae	Apamea remissa	7580662	7580662	2
Noctuidae	Apamea rubrirena	7558858	7558858	0
Noctuidae	Apamea scolopacina	6562476	6982476	1
Noctuidae	Apamea sordens	7580662	7580662	0
Noctuidae	Apamea sublustris	6914655	6914655	0
Noctuidae	Apamea unanimis	6911078	6982078	0
Noctuidae	Apamea zeta	7670605	7670605	0
Noctuidae	Aporophyla lutulenta	6600439	6775439	2
Noctuidae	Archanara algae	6914655	6914655	0
Noctuidae	Archanara dissoluta	6600439	6911439	1
Noctuidae	Archanara geminipuncta	6460852	6600852	1
Noctuidae	Archanara neurica	.	6268311	1
Noctuidae	Archanara sparganii	6550795	6770795	0
Noctuidae	Arenostola phragmitidis	6726787	6775787	0
Noctuidae	Asteroscopus sphinx	6591678	6591678	0
Noctuidae	Atethmia centrago	.	6359033	1
Noctuidae	Athetis gluteosa	6434180	6924180	0
Noctuidae	Athetis pallustris	7670605	7670605	1
Noctuidae	Autographa bractea	7260962	7575962	0
Noctuidae	Autographa buraetica	7260962	7260962	0
Noctuidae	Autographa excelsa	.	7580662	0
Noctuidae	Autographa gamma	7670605	7670605	0
Noctuidae	Autographa jota	6982210	7259210	0

Noctuidae	Autographa macrogamma	7670605	7670605	0
Noctuidae	Autographa mandarina	6460852	7020852	0
Noctuidae	Autographa pulchrina	7580662	7580662	0
Noctuidae	Axylia putris	6914655	6914655	0
Noctuidae	Blepharita amica	.	6726787	0
Noctuidae	Brachionycha nubeculosa	7580662	7580662	0
Noctuidae	Brachylomia viminalis	7580662	7580662	0
Noctuidae	Bryophila domestica	6543786	6543786	0
Noctuidae	Bryophila ereptricula	6268311	6268311	0
Noctuidae	Bryophila raptricula	6359033	6674033	0
Noctuidae	Bryophila ravula	.	6562476	0
Noctuidae	Calamia tridens	6726787	6775787	0
Noctuidae	Callopistria juventina	.	6395244	0
Noctuidae	Calophasia lunula	6914655	6982655	0
Noctuidae	Caradrina morpheus	7670605	7670605	1
Noctuidae	Celaena haworthii	7580662	7674662	0
Noctuidae	Celaena leucostigma	7259242	7580242	2
Noctuidae	Cerapteryx graminis	7670605	7670605	0
Noctuidae	Cerastis leucographa	6460852	6460852	0
Noctuidae	Cerastis rubricosa	7580662	7580662	0
Noctuidae	Charanyca trigrammica	6726787	6726787	0
Noctuidae	Chersotis cuprea	7580662	7580662	0
Noctuidae	Chilodes maritima	7580662	7580662	2
Noctuidae	Chloantha hyperici	6726787	6726787	0
Noctuidae	Chortodes elymi	7580662	7580662	0
Noctuidae	Chortodes extrema	6726787	6726787	0
Noctuidae	Chortodes fluxa	7580662	7580662	1
Noctuidae	Chortodes morrisii	6268311	6268311	0
Noctuidae	Chrysodeixis chalcites	6135149	6645149	0
Noctuidae	Coenobia rufa	6268311	6395311	0
Noctuidae	Coenophila subrosea	7580662	7580662	0
Noctuidae	Colocasia coryli	7580662	7580662	1
Noctuidae	Conisania leineri	6268311	6272311	0
Noctuidae	Conisania luteago	.	6309584	0
Noctuidae	Conistra erythrocephala	6726787	6770787	2
Noctuidae	Conistra rubiginea	6775458	6914458	0
Noctuidae	Conistra rubiginosa	6726787	6914787	0
Noctuidae	Conistra vaccinii	7259242	7259242	2
Noctuidae	Coranarta cordigera	7670605	7670605	0
Noctuidae	Cosmia affinis	6268311	6434311	0
Noctuidae	Cosmia diffinis	6434180	6434180	0
Noctuidae	Cosmia pyralina	6911078	6911078	0
Noctuidae	Cosmia trapezina	7225141	7581141	2
Noctuidae	Craniophora ligustri	6911078	6911078	1
Noctuidae	Cryphia algae	.	6460852	1
Noctuidae	Crypsedra gemmea	7580662	7580662	0
Noctuidae	Cryptocala chardinyi	.	7260962	1
Noctuidae	Cucullia absinthii	6726787	6876787	0

Noctuidae	Cucullia argentea	6434180	6434180	0
Noctuidae	Cucullia artemisiae	6460852	6460852	0
Noctuidae	Cucullia asteris	6550795	6600795	0
Noctuidae	Cucullia boryphora	.	6395244	0
Noctuidae	Cucullia chamomillae	6726787	6775787	0
Noctuidae	Cucullia fraudatrix	6460852	6460852	0
Noctuidae	Cucullia gnaphalii	6914655	6914655	0
Noctuidae	Cucullia lactucae	6914655	6914655	0
Noctuidae	Cucullia lucifuga	7225141	7225141	0
Noctuidae	Cucullia praecana	6268311	6276311	0
Noctuidae	Cucullia tanaceti	6268311	6268311	0
Noctuidae	Cucullia umbratica	7148468	7148468	0
Noctuidae	Dasypolia templi	7670605	7670605	0
Noctuidae	Deltote bankiana	6359033	6593033	0
Noctuidae	Deltote deceptoria	6460852	6460852	0
Noctuidae	Deltote uncula	7580662	7580662	0
Noctuidae	Diachrysia chrysitis	7259242	7259242	0
Noctuidae	Diachrysia chryson	.	6268311	0
Noctuidae	Diachrysia tutti	7259242	7259242	0
Noctuidae	Diarsia brunnea	7148468	7148468	0
Noctuidae	Diarsia dahlii	7580662	7710662	1
Noctuidae	Diarsia florida	6828249	6828249	0
Noctuidae	Diarsia mendica	7670605	7670605	2
Noctuidae	Diarsia rubi	7580662	7580662	1
Noctuidae	Dichonia aprilina	6911078	6911078	0
Noctuidae	Dicycla oo	6726787	6726787	1
Noctuidae	Diloba caeruleocephala	6914655	6914655	0
Noctuidae	Discestra melanopa	7670605	7670605	0
Noctuidae	Discestra trifolii	7259242	7259242	1
Noctuidae	Dryobotodes eremita	6726787	6910787	1
Noctuidae	Dypterygia scabriuscula	7259242	7259242	0
Noctuidae	Elaphria venustula	6460852	6910852	0
Noctuidae	Emmelia trabealis	6359033	6543033	0
Noctuidae	Enargia paleacea	7580662	7580662	0
Noctuidae	Epilecta linogrisea	6726787	6726787	0
Noctuidae	Epipsilia grisescens	6911078	6911078	0
Noctuidae	Eremobia ochroleuca	6460852	6600852	0
Noctuidae	Eremobina pabulatricula	6359033	6525033	0
Noctuidae	Eriopygodes imbecilla	6914655	6914655	0
Noctuidae	Eucarta virgo	.	6726787	0
Noctuidae	Eugnorisma depunctum	7259242	7259242	0
Noctuidae	Eugnorisma glareosum	6600439	6726439	1
Noctuidae	Eugraphe sigma	6562476	6600476	0
Noctuidae	Euplexia lucipara	7558858	7558858	0
Noctuidae	Eupsilia transversa	6982210	7259210	1
Noctuidae	Eurois occulta	7670605	7670605	1
Noctuidae	Euxoa adumbrata	6434180	6434180	1
Noctuidae	Euxoa cursoria	6726787	7576787	2

Noctuidae	Euxoa nigricans	7580662	7580662	0
Noctuidae	Euxoa obelisca	6911078	6982078	0
Noctuidae	Euxoa ochrogaster	6726787	6726787	2
Noctuidae	Euxoa recussa	7580662	7580662	1
Noctuidae	Euxoa tritici	7259242	7580242	2
Noctuidae	Euxoa vitta	6434180	6434180	0
Noctuidae	Gortyna flavago	6726787	7580787	1
Noctuidae	Graphiphora augur	7580662	7580662	0
Noctuidae	Hada plebeja	7670605	7670605	1
Noctuidae	Hadena albimacula	6911078	6982078	0
Noctuidae	Hadena bicruris	7259242	7259242	0
Noctuidae	Hadena caesia	6987888	6987888	0
Noctuidae	Hadena capsincola	6987888	6987888	0
Noctuidae	Hadena compta	6460852	6726852	0
Noctuidae	Hadena confusa	7259242	7259242	0
Noctuidae	Hadena filograna	6726787	6726787	0
Noctuidae	Hadena irregularis	6460852	6460852	0
Noctuidae	Hadena perplexa	7148468	7148468	0
Noctuidae	Hecatera bicolorata	7580662	7580662	2
Noctuidae	Hecatera dysodea	6268311	6748311	1
Noctuidae	Helicoverpa armigera	6268311	6977311	1
Noctuidae	Heliothis maritima	6434180	6434180	0
Noctuidae	Heliothis peltigera	6359033	6443033	0
Noctuidae	Heliothis viriplaca	6914655	6914655	0
Noctuidae	Hillia iris	7670605	7670605	0
Noctuidae	Hoplodrina ambigua	6268311	6771311	0
Noctuidae	Hoplodrina blanda	7148468	7259468	0
Noctuidae	Hoplodrina octogenaria	7225141	7225141	0
Noctuidae	Hydraecia micacea	7670605	7670605	1
Noctuidae	Hydraecia nordstroemi	6726787	7258787	1
Noctuidae	Hydraecia petasitis	6268311	6558311	0
Noctuidae	Hydraecia ultima	6460852	6726852	0
Noctuidae	Hyppa rectilinea	7670605	7670605	1
Noctuidae	Hyssia cavernosa	.	6359033	0
Noctuidae	Ipimorpha retusa	6914655	6914655	0
Noctuidae	Ipimorpha subtusa	6914655	7225655	0
Noctuidae	Jodia croceago	6268311	6268311	0
Noctuidae	Lacanobia contigua	7259242	7259242	0
Noctuidae	Lacanobia oleracea	7580662	7580662	0
Noctuidae	Lacanobia splendens	.	6434180	0
Noctuidae	Lacanobia suasa	7580662	7580662	2
Noctuidae	Lacanobia thalassina	7670605	7670605	0
Noctuidae	Lacanobia w-latinum	7580662	7580662	0
Noctuidae	Lamprotes c-aureum	6268311	6452311	0
Noctuidae	Lasionycta leucocycla	7670605	7670605	0
Noctuidae	Lasionycta proxima	7580662	7580662	0
Noctuidae	Lasionycta secedens	7670605	7670605	0
Noctuidae	Lasionycta skraelingia	7558858	7669858	0

Noctuidae	Lasionycta staudingeri	7670605	7670605	0
Noctuidae	Lithomoia solidaginis	7670605	7670605	1
Noctuidae	Lithophane consocia	7580662	7580662	0
Noctuidae	Lithophane furcifera	7580662	7580662	0
Noctuidae	Lithophane lamda	7580662	7580662	0
Noctuidae	Lithophane ornitopus	6726787	6770787	1
Noctuidae	Lithophane socia	7580662	7580662	0
Noctuidae	Litophane semibrunnea	.	6395244	0
Noctuidae	Luperina testacea	6911078	6911078	1
Noctuidae	Luperina zollikoferi	6591678	6789678	1
Noctuidae	Lycophotia porphyrea	7580662	7580662	0
Noctuidae	Macdunnoughia confusa	7558858	7558858	0
Noctuidae	Mamestra brassicae	7580662	7580662	1
Noctuidae	Melanchra persicariae	6600439	6775439	1
Noctuidae	Melanchra pisi	7670605	7670605	1
Noctuidae	Mesapamea secalella	6770907	7095907	2
Noctuidae	Mesapamea secalis	7148468	7148468	2
Noctuidae	Mesogona oxalina	6562476	6600476	0
Noctuidae	Mesoligia furuncula	6982210	6982210	1
Noctuidae	Mesoligia literosa	6914655	7259655	1
Noctuidae	Mniotype adusta	7580662	7580662	0
Noctuidae	Mniotype bathensis	.	6359033	0
Noctuidae	Mniotype satura	7580662	7580662	0
Noctuidae	Mniotype solieri	.	6268311	0
Noctuidae	Moma alpium	6726787	6726787	0
Noctuidae	Mythimna albipuncta	6460852	6610852	0
Noctuidae	Mythimna comma	7580662	7580662	0
Noctuidae	Mythimna conigera	7259242	7574242	0
Noctuidae	Mythimna ferrago	7148468	7148468	0
Noctuidae	Mythimna flammea	6600439	6675439	1
Noctuidae	Mythimna impura	7580662	7580662	0
Noctuidae	Mythimna l-album	.	6359033	0
Noctuidae	Mythimna languida	.	6359033	1
Noctuidae	Mythimna litoralis	6395244	6395244	0
Noctuidae	Mythimna obsoleta	7580662	7580662	0
Noctuidae	Mythimna pallens	7580662	7580662	1
Noctuidae	Mythimna pudorina	6550795	6770795	0
Noctuidae	Mythimna straminea	6770907	6775907	0
Noctuidae	Mythimna turca	6675586	6675586	0
Noctuidae	Mythimna unipuncta	.	6268311	0
Noctuidae	Mythimna vitellina	6268311	6688311	0
Noctuidae	Naenia typica	7580662	7580662	0
Noctuidae	Noctua comes	6726787	6987787	1
Noctuidae	Noctua fimbriata	6911078	7256078	2
Noctuidae	Noctua interjecta	6460852	6750852	1
Noctuidae	Noctua interposita	.	6637161	1
Noctuidae	Noctua janthe	6637161	6871161	1
Noctuidae	Noctua janthina	6460852	6644852	0

Noctuidae	Noctua orbona	6775458	6914458	1
Noctuidae	Noctua pronuba	7580662	7580662	2
Noctuidae	Nonagria typhae	7580662	7580662	2
Noctuidae	Nyctobrya muralis	.	6268311	1
Noctuidae	Ochropleura plecta	7580662	7580662	0
Noctuidae	Oligia fasciuncula	6562476	6688476	0
Noctuidae	Oligia latruncula	7259242	7259242	1
Noctuidae	Oligia strigilis	7148468	7259468	1
Noctuidae	Oligia versicolor	6268311	6461311	1
Noctuidae	Opigena polygona	7259242	7259242	0
Noctuidae	Orthosia cerasi	6911078	6911078	0
Noctuidae	Orthosia cruda	6914655	6914655	1
Noctuidae	Orthosia gothica	7670605	7670605	2
Noctuidae	Orthosia gracilis	6914655	6914655	0
Noctuidae	Orthosia incerta	7148468	7260468	2
Noctuidae	Orthosia miniosa	6584475	6775475	1
Noctuidae	Orthosia opima	7580662	7580662	0
Noctuidae	Orthosia populeti	6914655	7259655	1
Noctuidae	Pachetra sagittigera	6600439	6726439	0
Noctuidae	Panemeria tenebrata	6264029	6264029	0
Noctuidae	Panolis flammea	7580662	7580662	0
Noctuidae	Panthea coenobita	6911078	6911078	0
Noctuidae	Papestra biren	7670605	7670605	0
Noctuidae	Paradrina clavipalpis	7259242	7261242	1
Noctuidae	Paradrina selini	7580662	7580662	0
Noctuidae	Parastichtis suspecta	7670605	7670605	1
Noctuidae	Parastichtis ypsillon	7225141	7375141	1
Noctuidae	Peridroma saucia	6268311	6782311	2
Noctuidae	Phlogophora meticulosa	6726787	6914787	1
Noctuidae	Photedes captiuncula	6914655	6914655	0
Noctuidae	Photedes minima	7580662	7650662	0
Noctuidae	Phragmatiphila nexa	6675586	6726586	0
Noctuidae	Platyperigea montana	7259242	7580242	0
Noctuidae	Plusia festucae	7580662	7580662	0
Noctuidae	Plusia putnami	7259242	7580242	0
Noctuidae	Polia bombycina	7225141	7302141	1
Noctuidae	Polia conspicua	.	7580662	0
Noctuidae	Polia hepatica	7580662	7580662	0
Noctuidae	Polia lamuta	7670605	7670605	0
Noctuidae	Polia nebulosa	6911078	7222078	0
Noctuidae	Polia richardsoni	7670605	7670605	0
Noctuidae	Polychrysia moneta	7580662	7580662	0
Noctuidae	Polymixis flavicincta	6550795	6550795	0
Noctuidae	Polymixis polymita	6914655	6914655	0
Noctuidae	Protarchanara brevilinea	.	6268311	0
Noctuidae	Protodeltote pygarga	6600439	6861439	0
Noctuidae	Protolampra sobrina	7580662	7671662	0
Noctuidae	Proxenus lepigone	7580662	7580662	0

Noctuidae	Pseudeustrotia candidula	6726787	6775787	0
Noctuidae	Pseudohadena immunda	7225141	7225141	0
Noctuidae	Pyrrhia umbra	6914655	7148655	0
Noctuidae	Rhizedra lutosa	7580662	7580662	1
Noctuidae	Rhyacia simulans	7148468	7148468	0
Noctuidae	Rusina ferruginea	7259242	7580242	0
Noctuidae	Schinia scutosa	6726787	7103787	0
Noctuidae	Sedina buettneri	6268311	6600311	0
Noctuidae	Sedina pygmina	7580662	7580662	1
Noctuidae	Shargacucullia lychnitis	6460852	6460852	0
Noctuidae	Shargacucullia scrophulariae	6460852	6460852	0
Noctuidae	Shargacucullia verbasci	6600439	6600439	0
Noctuidae	Sideridis reticulata	7259242	7259242	0
Noctuidae	Sideridis rivularis	7670605	7670605	0
Noctuidae	Sideridis turbida	6726787	6726787	0
Noctuidae	Simyra albovenosa	7580662	7580662	1
Noctuidae	Spaelotis clandestina	7580662	7580662	0
Noctuidae	Spaelotis ravida	7259242	7259242	0
Noctuidae	Spodoptera exigua	6726787	6726787	0
Noctuidae	Spodoptera littoralis	.	6359033	0
Noctuidae	Standfussiana lucernea	7580662	7580662	0
Noctuidae	Staurophora celsia	7259242	7580242	0
Noctuidae	Sympistis funebris	7670605	7670605	0
Noctuidae	Sympistis heliophila	7670605	7670605	0
Noctuidae	Sympistis lapponica	7670605	7670605	0
Noctuidae	Sympistis nigrita	7670605	7670605	0
Noctuidae	Syngrapha diasema	7670605	7670605	0
Noctuidae	Syngrapha hochenwarthi	7670605	7670605	0
Noctuidae	Syngrapha interrogationis	7670605	7670605	0
Noctuidae	Syngrapha microgamma	7670605	7670605	0
Noctuidae	Syngrapha parilis	7670605	7670605	0
Noctuidae	Thalpophila matura	6775458	6775458	1
Noctuidae	Tholera cespitis	7580662	7580662	0
Noctuidae	Tholera decimalis	7360639	7360639	0
Noctuidae	Tiliacea sulphurago	6268311	6268311	0
Noctuidae	Trachea atriplicis	7148468	7148468	0
Noctuidae	Trichoplusia ni	.	6600439	0
Noctuidae	Trichosea ludifica	6268311	6268311	0
Noctuidae	Tyta luctuosa	7148468	7438468	0
Noctuidae	Victrix umovii	6726787	7096787	0
Noctuidae	Xanthia aurago	6726787	6775787	2
Noctuidae	Xanthia citrago	6911078	6911078	1
Noctuidae	Xanthia gilvago	6726787	6775787	1
Noctuidae	Xanthia icteritia	7670605	7670605	1
Noctuidae	Xanthia ocellaris	6268311	6600311	1
Noctuidae	Xanthia togata	7670605	7670605	0
Noctuidae	Xestia alpicola	7670605	7670605	0
Noctuidae	Xestia ashworthii	6770907	6987907	2

Noctuidae	Xestia atrata	.	6987888	0
Noctuidae	Xestia baja	7259242	7580242	0
Noctuidae	Xestia borealis	7670605	7670605	0
Noctuidae	Xestia castanea	6726787	6775787	1
Noctuidae	Xestia c-nigrum	7580662	7580662	1
Noctuidae	Xestia collina	6911078	6993078	0
Noctuidae	Xestia distensa	7670605	7670605	0
Noctuidae	Xestia ditrapezium	6268311	6408311	0
Noctuidae	Xestia fennica	7670605	7670605	0
Noctuidae	Xestia gelida	7670605	7670605	0
Noctuidae	Xestia laetabilis	7558858	7698858	0
Noctuidae	Xestia lorezi	7670605	7670605	0
Noctuidae	Xestia lyngei	7670605	7670605	0
Noctuidae	Xestia quieta	7670605	7670605	0
Noctuidae	Xestia sexstrigata	6982210	7148210	0
Noctuidae	Xestia sincera	7580662	7580662	0
Noctuidae	Xestia speciosa	7670605	7670605	1
Noctuidae	Xestia stigmatica	6911078	6911078	0
Noctuidae	Xestia tecta	7670605	7670605	0
Noctuidae	Xestia triangulum	6982210	7148210	0
Noctuidae	Xestia xanthographa	6982210	7259210	2
Noctuidae	Xylena exsoleta	6911078	6911078	0
Noctuidae	Xylena vetusta	7670605	7670605	0
Noctuidae	Xylocampa areola	6562476	6562476	0

Supplementary References

1 Hickling, R., Roy, D. B., Hill, J. K., Fox, R. & Thomas, C. D. The distributions of a wide range of taxonomic groups are expanding polewards. *Global Change Biology* **12**, 450-455, doi:10.1111/j.1365-2486.2006.01116.x (2006).

2 Pöyry, J., Luoto, M., Heikkinen, R. K., Kuussaari, M. & Saarinen, K. Species traits explain recent range shifts of Finnish butterflies. *Global Change Biology* **15**, 732-743, doi:10.1111/j.1365-2486.2008.01789.x (2009).

3 Devictor, V. *et al.* Differences in the climatic debts of birds and butterflies at a continental scale. *Nature Clim. Change* **2**, 121-124, doi:http://www.nature.com/nclimate/journal/v2/n2/abs/nclimate1347.html#supplementary-information (2012).

4 Betzholtz, P.-E., Pettersson, L. B., Ryrholm, N. & Franzén, M. With that diet, you will go far: trait-based analysis reveals a link between rapid range expansion and a nitrogen-favoured diet. *Proc. R. Soc. B.* **280**, doi:10.1098/rspb.2012.2305 (2013).

5 Kwon, T.-S., Lee, C. & Kim, S.-S. Northward range shifts in Korean butterflies. *Climatic Change* **126**, 163-174, doi:10.1007/s10584-014-1212-2 (2014).

6 Aguirre-Gutiérrez, J. *et al.* Functional traits help to explain half-century long shifts in pollinator distributions. *Scientific Reports* **6**, 24451, doi:10.1038/srep24451 (2016).

7 Grewe, Y., Hof, C., Dehling, D. M., Brandl, R. & Brändle, M. Recent range shifts of European dragonflies provide support for an inverse relationship between habitat predictability and dispersal. *Global Ecol. Biogeogr.* **22**, 403-409, doi:10.1111/geb.12004 (2013).

8 Ogawa-Onishi, Y. & Berry, P. M. Ecological impacts of climate change in Japan: The importance of integrating local and international publications. *Biol. Conserv.* **157**, 361-371, doi:http://dx.doi.org/10.1016/j.biocon.2012.06.024 (2013).

9 Schönrogge, K. *et al.* Range expansion and enemy recruitment by eight alien gall wasp species in Britain. *Insect Conservation and Diversity* **5**, 298-311, doi:10.1111/j.1752-4598.2011.00161.x (2012).

10 Thomas, C. D. & Lennon, J. J. Birds extend their ranges northwards. *Nature* **399**, 123-213 (1999).

11 Auer, S. K. & King, D. I. Ecological and life-history traits explain recent boundary shifts in elevation and latitude of western North American songbirds. *Global Ecol. Biogeogr.* **23**, 867-875, doi:10.1111/geb.12174 (2014).

12 Brommer, J. E. The range margins of northern birds shift polewards. *Ann. Zool. Fennici* **41**, 391-397 (2004).

13 Hitch, A. T. & Leberg, P. L. Breeding distributions of North American bird species moving north as a result of climate change. *Conserv. Biol.* **21**, 534-539, doi:10.1111/j.1523-1739.2006.00609.x (2007).

14 Devictor, V., Julliard, R., Couvet, D. & Jiguet, F. Birds are tracking climate warming, but not fast enough. *Proc. R. Soc. B.* **275**, 2743-2748, doi:10.1098/rspb.2008.0878 (2008).

15 Zuckerberg, B., Woods, A. M. & Porter, W. F. Poleward shifts in breeding bird distributions in New York State. *Global Change Biology* **15**, 1866-1883, doi:10.1111/j.1365-2486.2009.01878.x (2009).

16 Brommer, J. E., Lehikoinen, A. & Valkama, J. The breeding ranges of central European and Arctic bird species move poleward. *PLoS ONE* **7**, e43648, doi:10.1371/journal.pone.0043648 (2012).

17 VanDerWal, J. *et al.* Focus on poleward shifts in species' distribution underestimates the fingerprint of climate change. *Nature Clim. Change* **3**, 239-243, doi:http://www.nature.com/nclimate/journal/v3/n3/abs/nclimate1688.html#supplementary-information (2013).

18 Gillings, S., Balmer, D. E. & Fuller, R. J. Directionality of recent bird distribution shifts and climate change in Great Britain. *Global Change Biology* **21**, 2155-2168, doi:10.1111/gcb.12823 (2015).

19 Moreno-Rueda, G., Pleguezuelos, J. M., Pizarro, M. & Montori, A. Northward shifts of the distributions of Spanish reptiles in association with climate change. *Conserv. Biol.* **26**, 278-283 (2011).
